# Supplementary material for: Hypoxia and ERα Transcriptional Crosstalk Is Associated with Endocrine Resistance in Breast Cancer
Source: Cancers (Basel). 2022 Oct 8;14(19):4934. doi: 10.3390/cancers14194934 (PMC9563995; doi:10.3390/cancers14194934)
Supplement: Supplementary file 1 [file cancers-14-04934-s001.zip › cancers-1929478-supplementary.pdf]

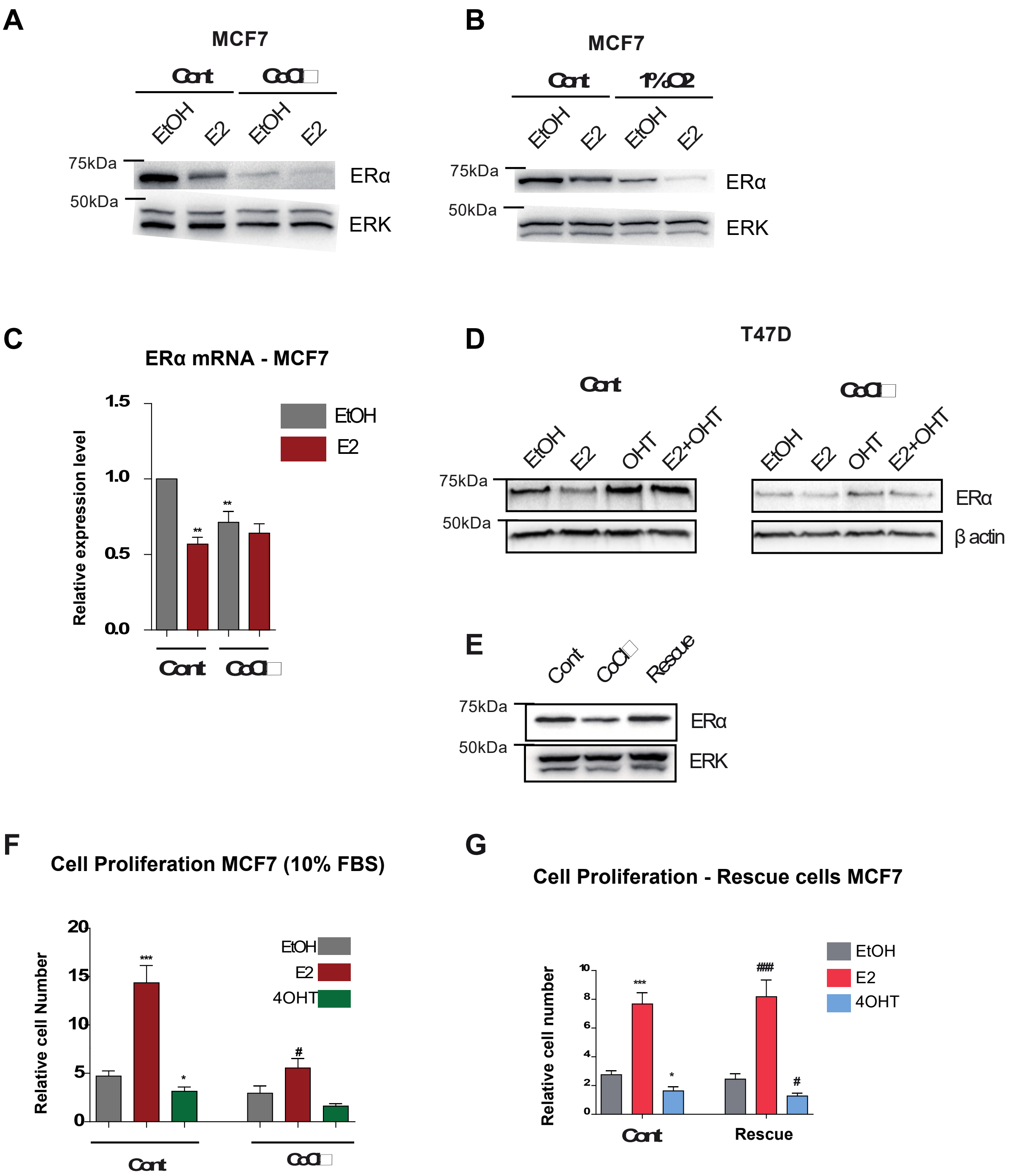

Figure S1. ER protein/mRNA expression and MCF7 cells proliferation under hypoxia. **(A)** Western-Blot showing ER protein expression level after 24 h of exposure to CoCl2, in presence or absence of 10 nM of E2 for 24 h. ERK1/2 was used as loading control. **(B)** Western-Blot showing ER protein expression level after 24h in presence of 1% O2 atmosphere using H35 Hypoxystation (Don Whitley), in presence or absence of 10 nM of E2 for 24 h. ERK1/2 was used as loading control. **(C)** qPCR showing ER mRNA expression level after prolonged exposure to CoCl2, in presence or absence of 10 nM of E2 for 24 h. **(D)** Western-Blots showing ER protein expression in T47D treated or not with CoCl2, in presence or absence of 10 nM of E2 or 1 uM of 4-OHT for 24 h. -actin was used as loading control. **(E)** Western blot showing ER protein abundance in MCF7 control

cells, CoCl2-treated cells (1 month) and in cells hypoxic cells for which CoCl2 was removed from the media for 1 month (Rescue). ERK was used as normalizing control. **(F)** Proliferation count under 10% FBS steroid free DMEM, of MCF7 treated for 6 days with EtOH or 10 nM E2 or 1 μM 4-OHT. Values are expressed in fold change compared to the number of seeded cells at the beginning of the treatment. **(G)** Proliferation of control and rescue (1-month hypoxic stress followed by 1-month of recovery in CoCl2 free medium) MCF7 treated for 6 days with EtOH, 10 nM E2 or 1 μM 4-OHT, in presence of 2 % of steroid-free FBS. Values are expressed in fold change compared to the number of seeded cells at the beginning of the treatment. *p-value < 0.05, **p-value < 0.01 and ***p-value < 0.001 with a Mann-Whitney test for comparisons against the control. #p-value < 0.05 with a Mann-Whitney test for comparisons against CoCl2 treatment.

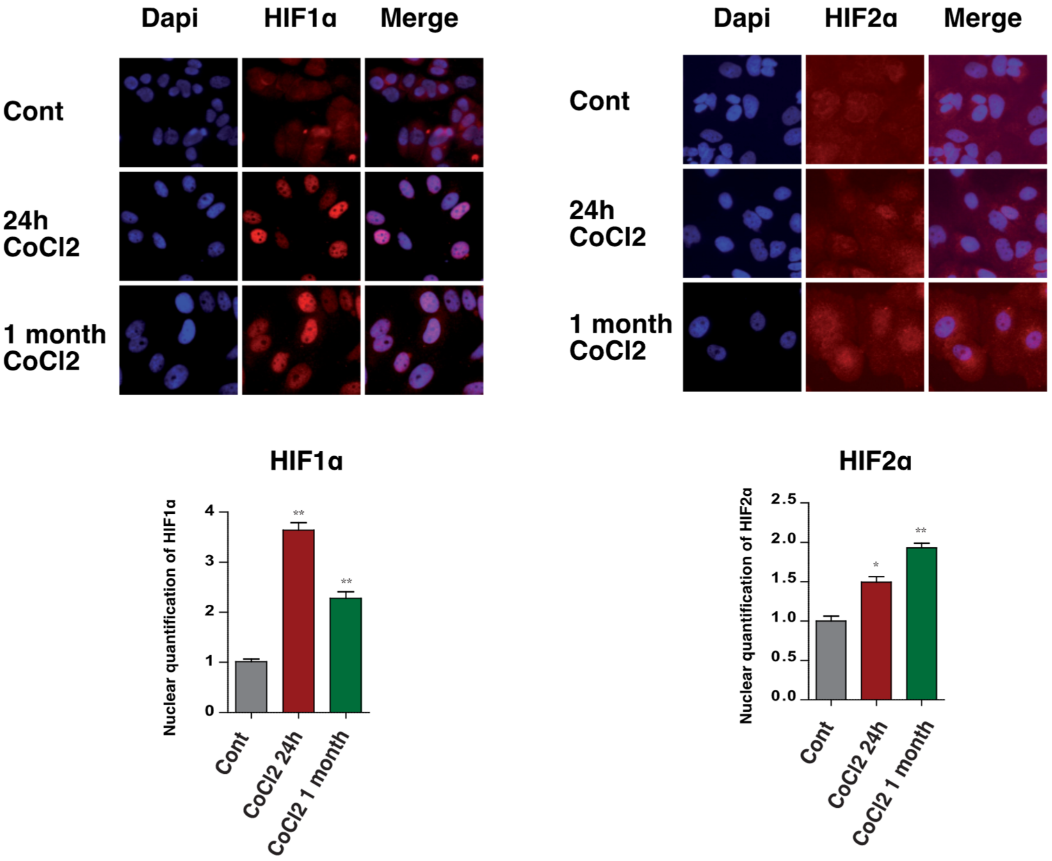

Figure S2. HIF1 and HIF2 expression and localization in MCF7 cells after CoCl2 treatment. Immunofluorescence experiments showing HIF1 and HIF2 nuclear localization upon CoCl2 exposure (24h or 1-month). Nuclear signal was quantified using Image J software. *p-value < 0.05, **p-value < 0.01 and ***p-value < 0.001 with a Mann-Whitney test for comparisons against the control.

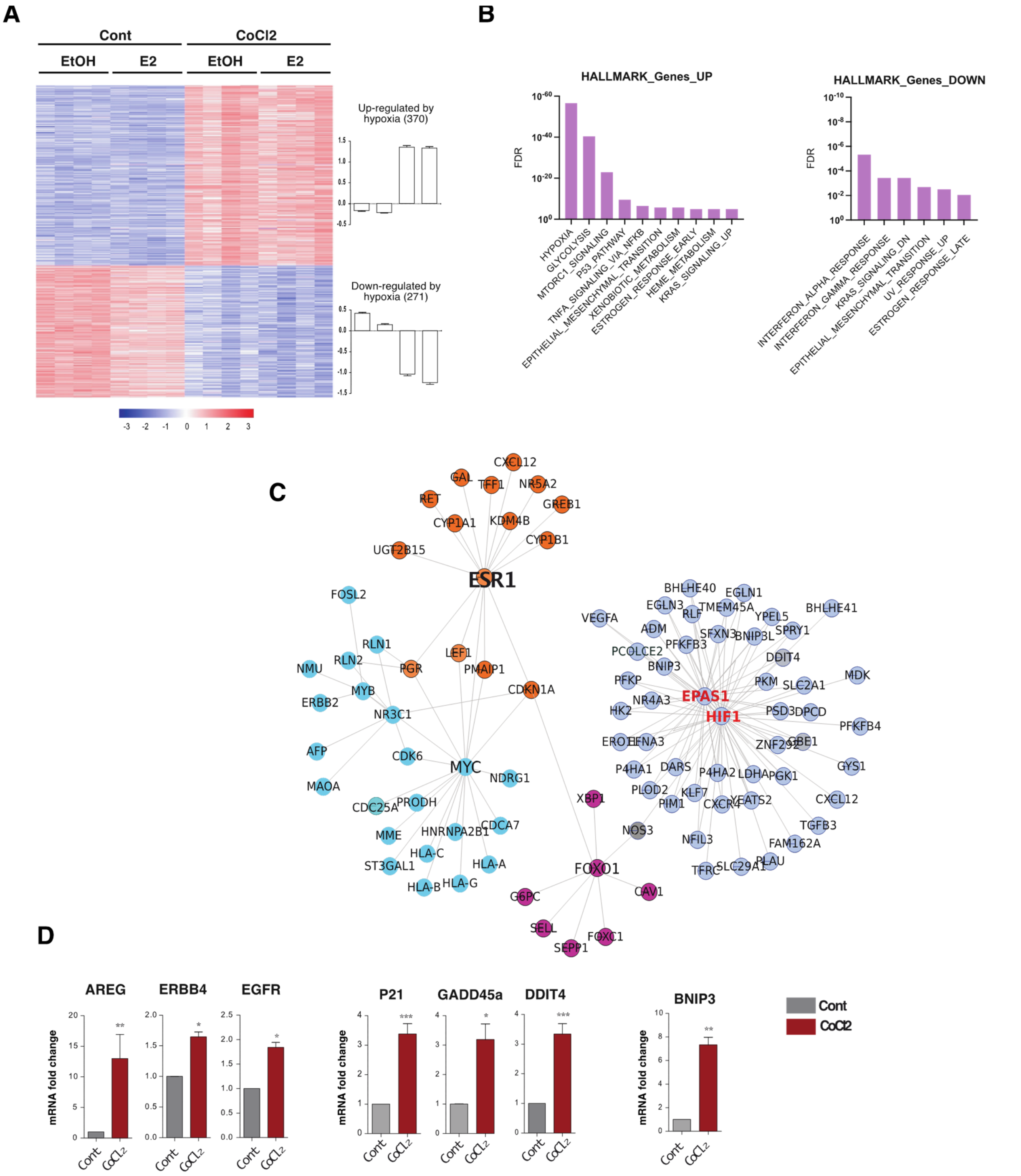

Figure S3. Characterization of the hypoxia mimicking agent CoCl2 response on MCF7 cells. (A) Differential expression analysis (FDR < 0.05 and FC > ±1.8) of hypoxia-regulated genes only. **(B)** Functional annotation of genes up and down regulated by CoCl2 treatment, using HALLMARK resources from GSEA (https://www.gsea-msigdb.org/gsea/msigdb/) **(C)** Transcriptional network built from transcript profiling data. Network was built using the AMEN software. HIF1 and HIF2 were added to network and used as central knots. **(D)** RT-qPCR experiments on hypoxia-regulated genes associated with endocrine resistance (*AREG, ERBB4, EGFR*), cell-cycle and cell-growth arrest (*P21, GADD45a, DDIT4*) and apoptosis (*BNIP3*). *p-value < 0.05, **p-value < 0.01 and ***p-value < 0.001 with a Mann-Whitney test for comparisons against control.

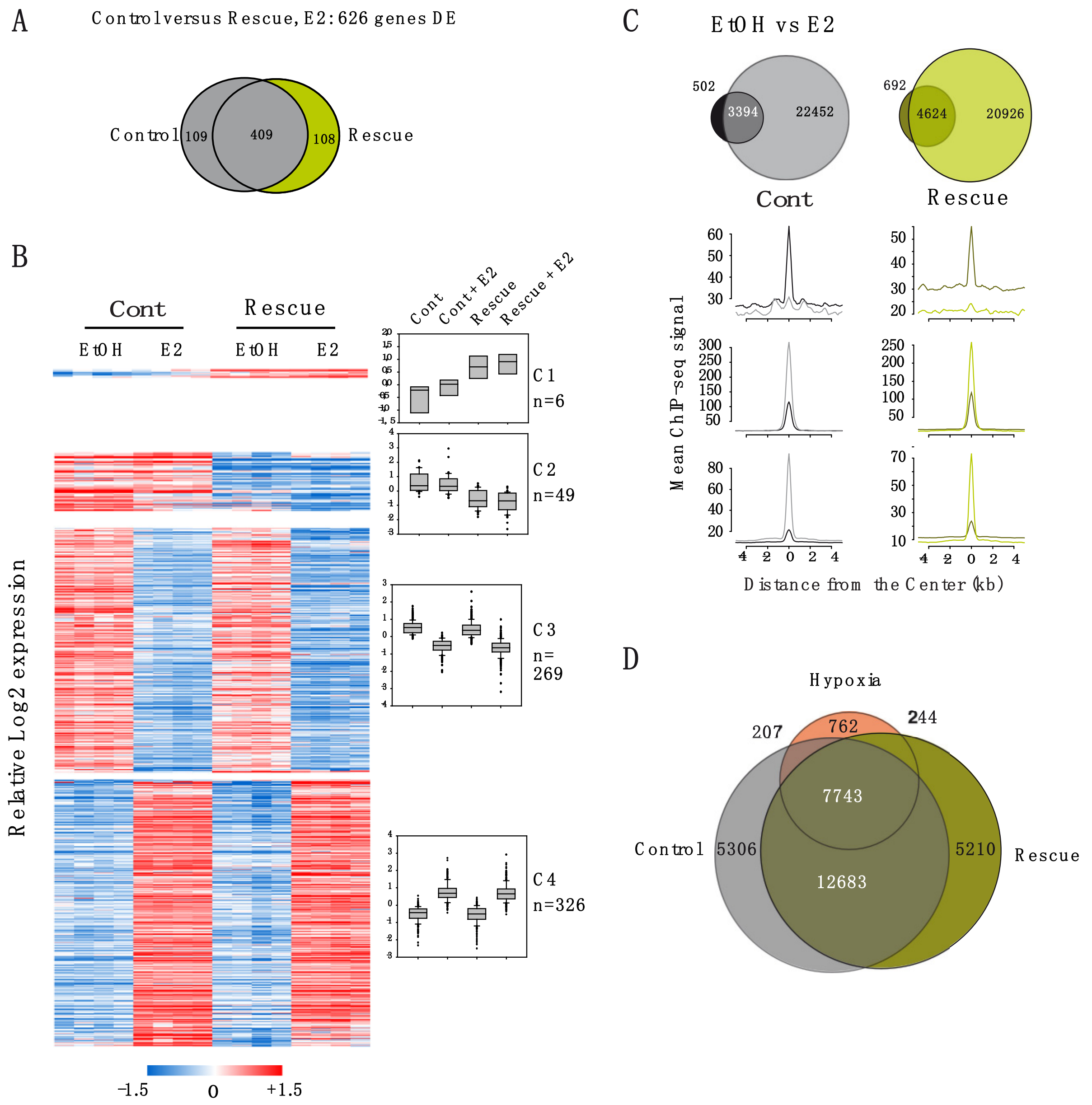

Figure S4. Cessation of hypoxia restores E2-mediated transcription and ER binding to chromatin genome wide. **(A)** & **(B)** Heatmap showing supervised clustering (k-means method) of total E2-regulated genes both in control and rescue (1-month hypoxic stress followed by 1-month of recovery in CoCl2 free medium) MCF7 cells (FC > 1.8 – FDR < 0.05). Control and CoCl2-treated MCF7 cells were treated with EtOH or 10 nM of E2. Transcriptomes from four independent biological samples analyzed by microarray. Graphs on the left represent the average of expression value (Log2) of all genes of each cluster. The number of genes in each cluster are indicated. **(C)** Venn diagrams showing ERBSs overlapping in E2 or EtOH-treated cells for 50 minutes, either in control or rescue MCF7 cells, as determined from ChIP-seq experiments. The ER ChIP-seq signals were aligned and averaged within a -2/+2 kbp window centered on ERBS belonging to the 3 categories in the upper Venn diagrams. **(D)** Venn diagram showing ERBSs overlapping between control, CoCl2-treated and rescued cells. All ERBSs detected in absence or presence of E2 were included in the analysis.

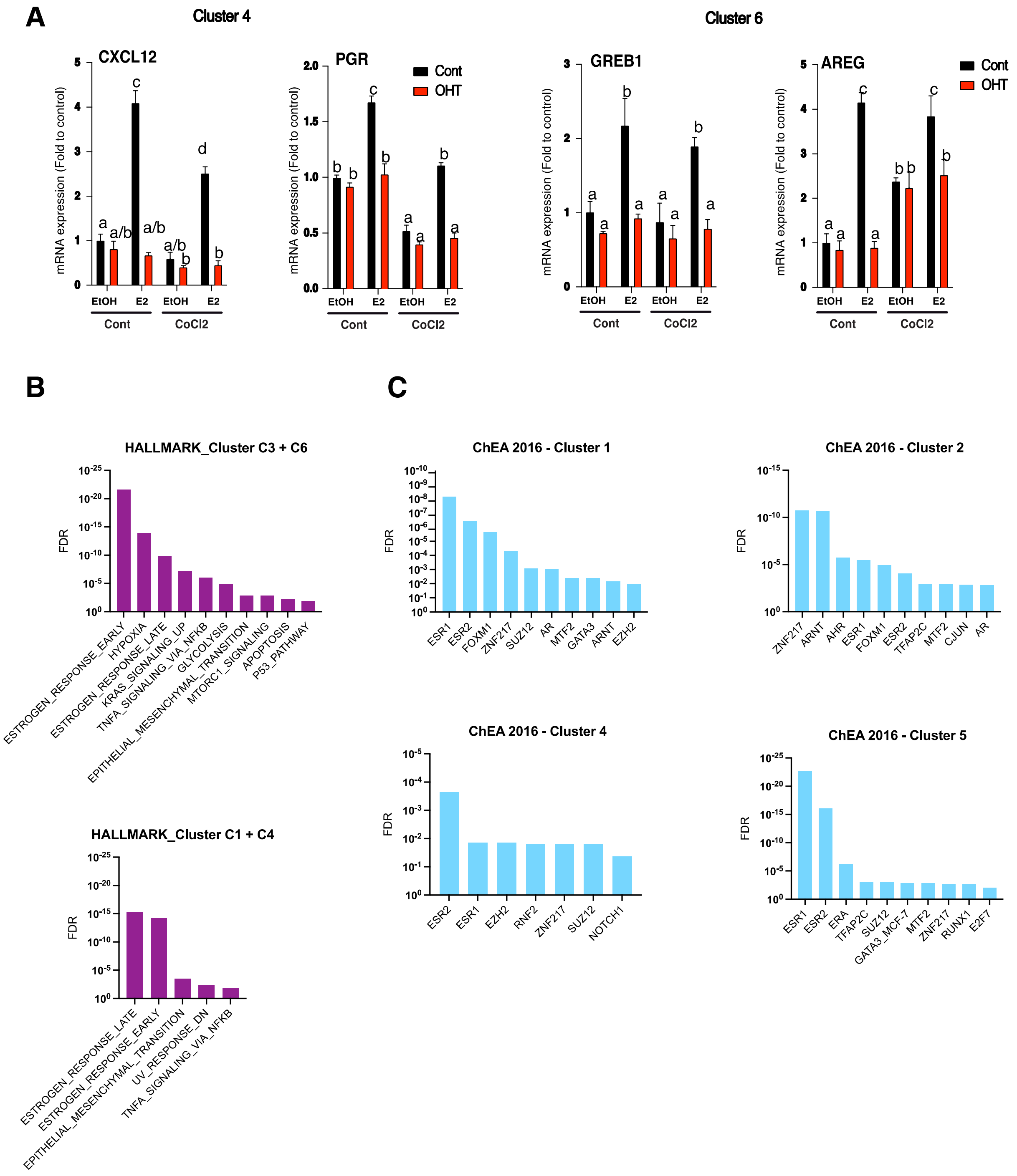

Figure S5. Effects of hypoxia on E2-response in T47D cell line and micro-array clusters functional annotation. (A) RT-qPCR showing *CXCL12*, *PGR*, *GREB1* and *AREG* expression level after 24 h exposure to CoCl2, in presence or absence of 10 nM of E2 and/or 1 uM 4-OHT for 24 h. Values represent the mean +/- SD of triplicate and are expressed as a fold induction above the value of untreated cells. (*p < 0.05, student 's t-test). **(B)** Functional annotation of genes belonging to clusters 3 and 6 together (E2 dependent – upregulated by CoCl2), and to clusters 1 and 4 together (E2 dependent – downregulated by CoCl2), using HALLMARK resources from GSEA (https://www.gsea-msigdb.org/gsea/msigdb/) **(C)** Analysis of transcription factors binding enrichment using ChEA algorithm on genes belonging to the clusters 1, 2, 4 and 5.

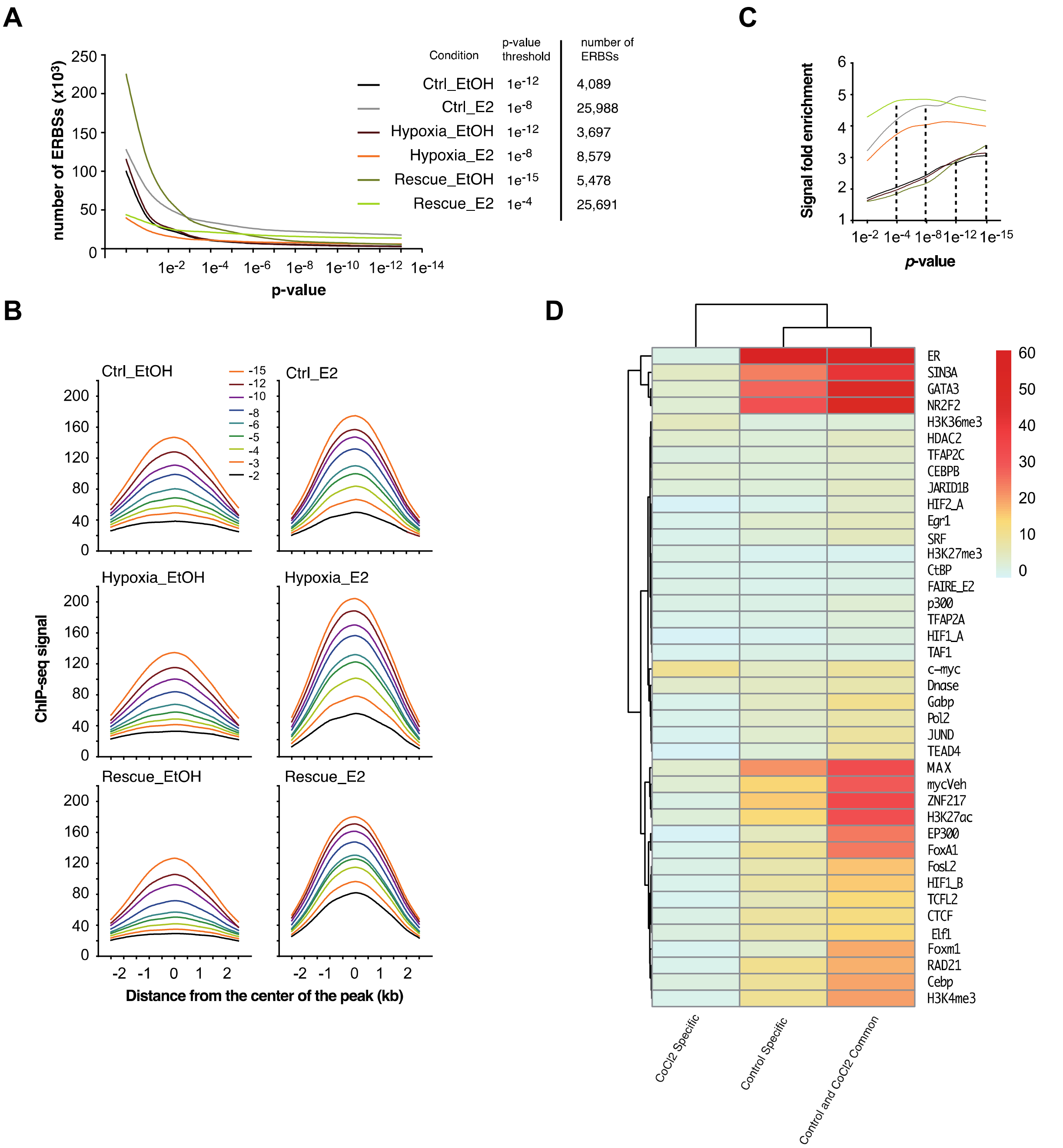

Figure S6. Determination of specific ER cistromes and transcription factor motif analysis. **(A)** We identified ER enriched regions (ERBSs) in MCF-7 cells treated or not with E2 for 50 min in presence or absence hypoxia mimetic agent CoCl2, using different thresholds of significance and peaks defined as being constituted of at least 4 adjacent signals within 65 bp above the threshold values. The number of ERBSs identified is shown here as a function of the p-value used. The table on the right side of the panel indicates the number of ERBSs at the chosen p-value found to have to best signal to noise ratio. **(B)** Each graph represents the mean ChIP-seq signal observed on the different sets of ERBSs determined in Panel A in a -2.5/+2.5 kb window centered at each ERBS summit. **(C)** The mean signal determined on all sets of ERBSs identified at each p-values was normalized to background signals measured at -5kb from the center of each ERBS. These normalized values indicate the mean fold enrichment of identified ERBSs in ER. We determined the best p-value threshold indicated in Panel A for discriminating the signal-to-noise within ERBSs as the one determining the plateau of these curves, as illustrated by the dotted line. **(D)** Hierarchical clustering of the percentage of overlap of ER

cistromes in control and CoCl2 treated cells with the binding sites of other transcription factors or enriched regions of histones marks previously determined in MCF7 cells. Analysis was made on CoCl2 specific ERBSs, control specific ERBSs and common ERBSs. References of published CHIP-Seq data are listed in supplementary table 3.

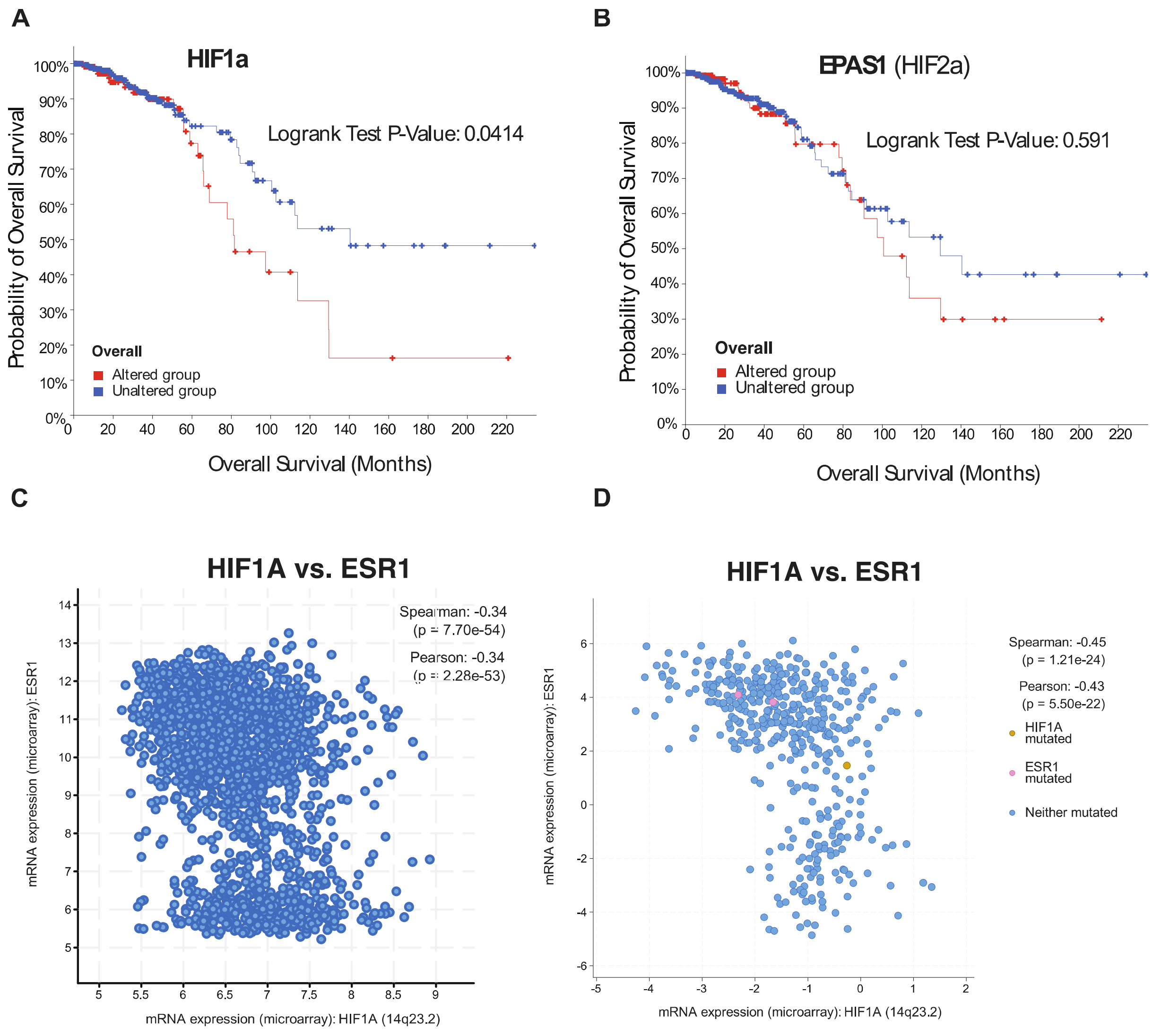

Figure S7. HIF1 and HIF2 clinical data and correlation with *ESR1*. (A) & (B) HIF1 and HIF2 mRNA expression prediction value regarding overall survival (OS) in TCGA cohort (z-score = 1, n = 463). **(C) & (B)** *HIF1a* and *ESR1* mRNA expression anti correlation in the Metabric (n = 1980) and TCGA (n = 460) cohorts, respectively. Spearman and Pearson correlation values and p-value are shown.

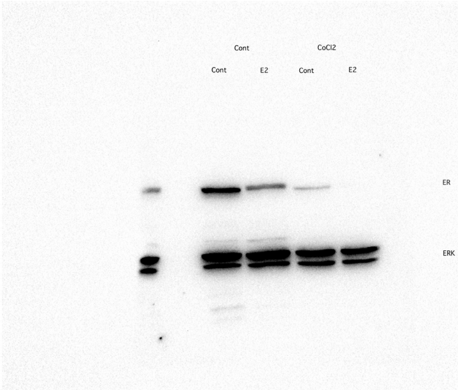

ER

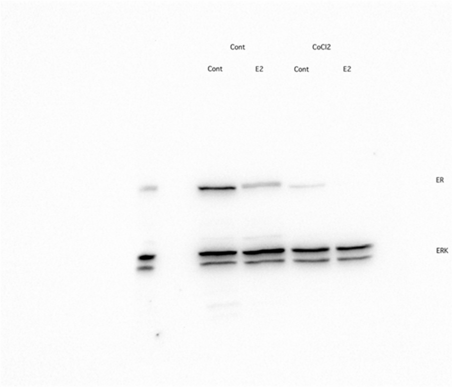

ERK

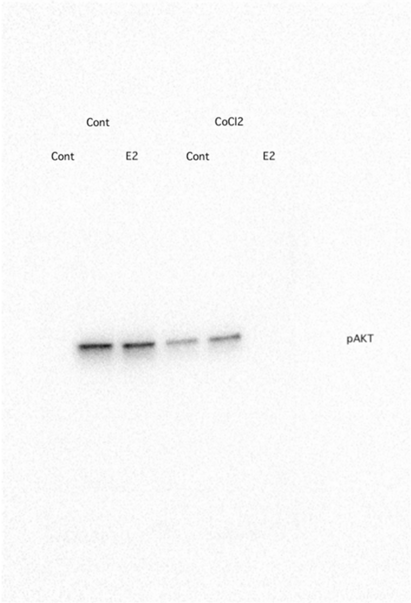

pAKT ser 473

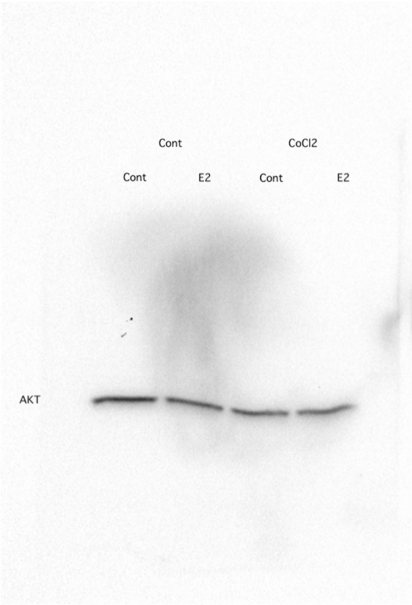

AKT total

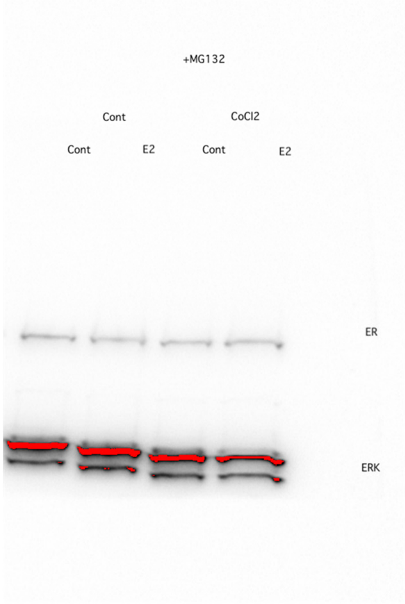

ER

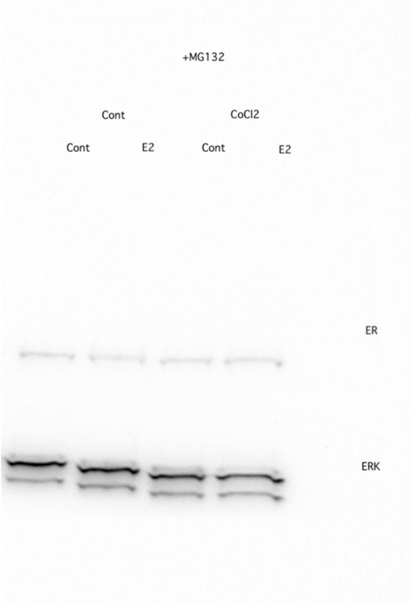

ERK

Figure S8. The original western blot figure of figure 1A.

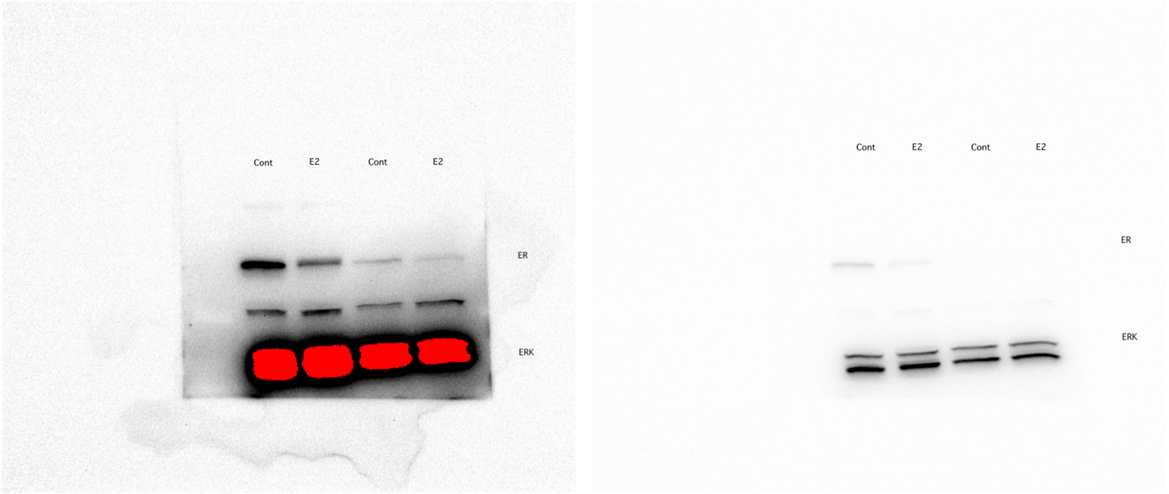

ER *EK*

Figure S9. The original western blot figure of figure S1A.

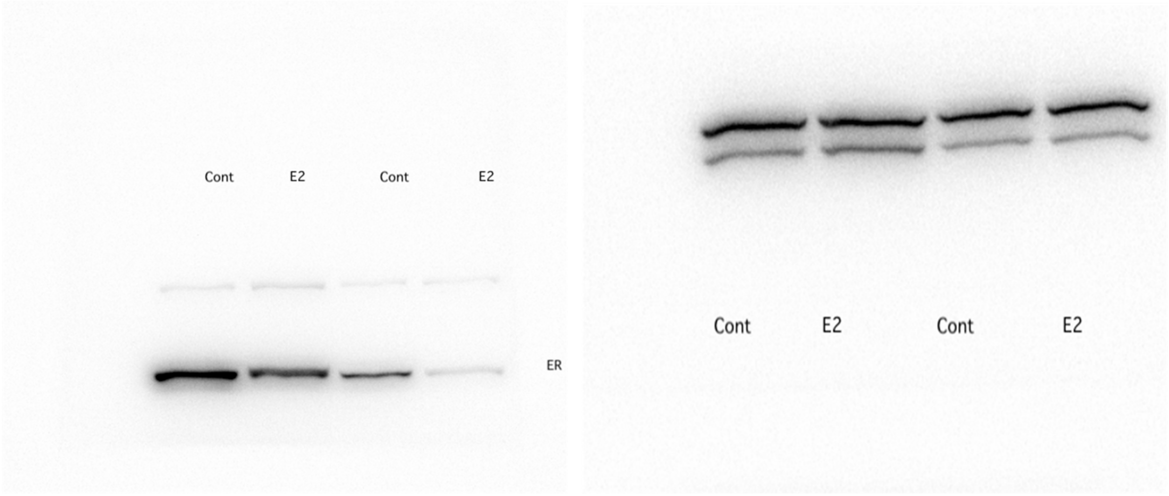

ER *ERK*

Figure S10. The original western blot figure of figure S1B.

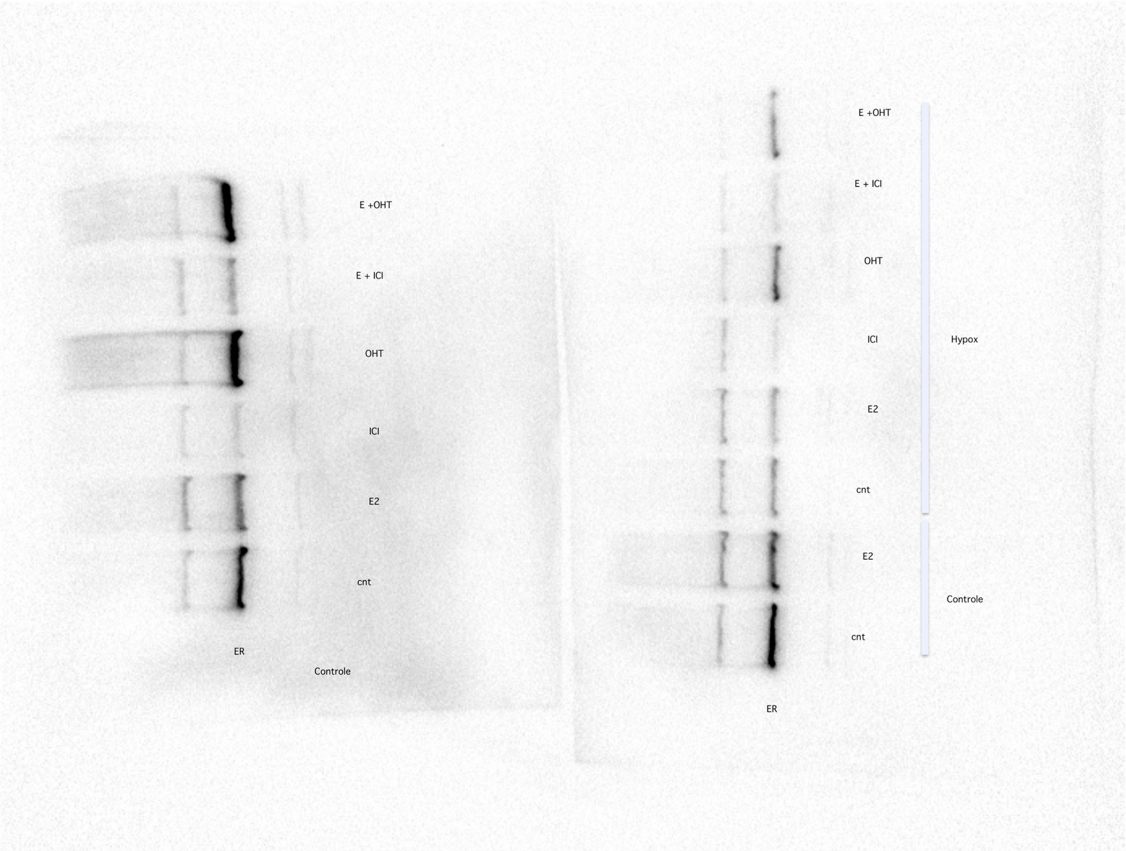
E +OHT
E + ICI
OHT
ICI
E2
cnt
ER
Controle
E +OHT
E + ICI
OHT
ICI
E2
cnt
Hypox
E2
cnt
Controle
ER

ER

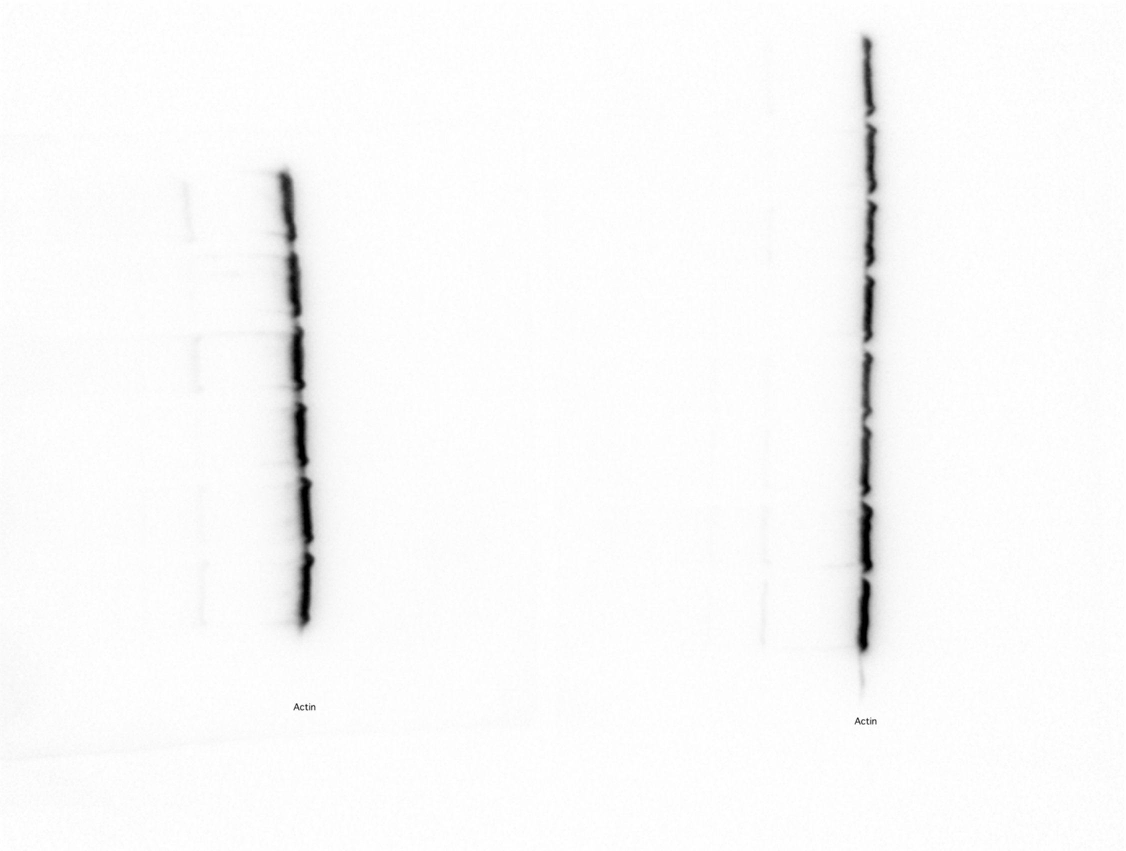
Actin
Actin

Actin

Figure S11. The original western blot figure of figure S1D.

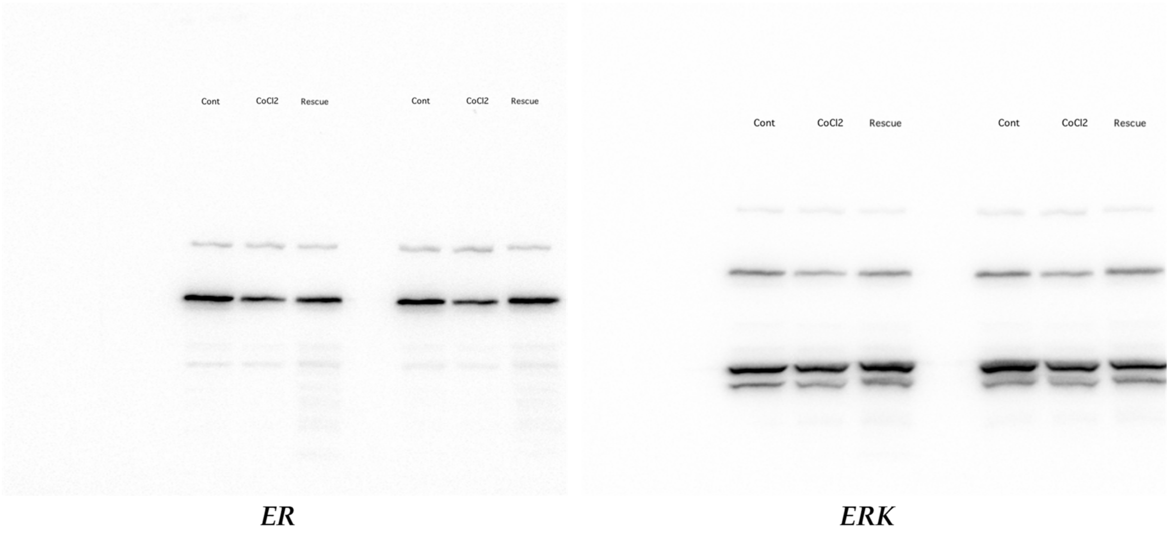

Figure S12. The original western blot figure of figure S1E.

Table S1. List of primers used in qPCR to quantify mRNA expression and CHIP.

RT-qPCR	Forward	Reverse
SGK1	GACAGGACTGTGGACTGGTG	TTTCAGCTGTGTTTCGGCTA
AREG	GTATTTTCACTTTCCGTCTTG	CCTGGGTATATTGTCGATTCA
CXCL12	CTCCTGGGGATGTGTAATGG	GCCTCCATGGCATACATAGG
TFF1	ACCATGGAGAACAAGGTGA	CCGAGCTCTGGGACTAATCA
PGR	GTGCCTATCCTGCCTCTCAATC	CCCGCCGTCGTAACTTTCG
GREB1	GAGGATGTGGAGTGGAGACC	CAGTACCTCAAAGACCTCGGC
TBP	AGGCAAGGGTACATGAGAGCCA	TGGCTCTCATGTACCCTTGCCT
DDIT4	AGGAAGCTCATTGAGTTGTG	GGTACATGCTACACACACAT
ERα	AGTCACAATGAACCTGCAAGC	GGCCAGGTCATTCTCCACATT
HCK	CTGCCAACATCTTGGTCTCT	TCAGCAGGATACCAAAGGAC
CDC25a	CAAACCTTGACAACCGATGC	ACACTGACCGAGTGCTGGAG
p21	TGTGGACCTGTCACTGTCTTG	TAGGGCTTCCTCTTGGAGAA
KDM4b	GACATCAGCGGCTCTTTGTATGATG	ATGCCGAAGTACAGGTAGGGCGTG
BNIP3	CAGGGCTCCTGGGTAGAACT	CTACTCCGTCCAGACTCATGC
EGFR	GTGACCGTTTGGGAGTTGATGA	GGCTGACGGAGGCGTTCTC
HIF1A	TCCAAGAAGCCCTAACGTGT	TGATCGTCTGGCTGCTGTAA
HIF2A	ACACACAAGCTCCTCTCCTC	AGCTCCACCTGTGTAAGTCC
**CHIP-qPCR**		
FOXC1	GCTGGTGGCACTCAGAAAGT	CCCTCCTTTGACAGGAAGAA
AREG	AGCTAGAAGCGTTTGCAGGA	TGGGTGTGTGTTTGTCCACT
CXCL12 ERE1	TGCTCCGCTAAGGTTTCAGT	GCACCTAGTCACACCCACCT
NTN1	GAACAAGGATGGAGGGTGAA	CCCCTCTACAAGGCAAGATG
GREB1	GCACACTTTCCACCTCTGCT	GCTCGCTTGCAAAGTCAAAT
TFF1	GTCGCACTTCTCGAAGGTCT	CAGGTAAGGCGTGCTTCTTC
FOX20 (internal control)	TTTGCTTTATTTGGCGCTTT	TGAGCGATTGCAAAGAGATG
HCK	GTAGGTGCCTGTGACCCTGT	TACTTCAGCCTCTGGCTGGT

KDM4B	TCTGATTGGACACCTGGCAG	GCTTCCACCAAAACAGCCTG
CDC25a	AGGCCTGAGAGATGGGAAGA	ACGGGTCATGCTGTTGTCTC
PGR	GCAGGACGACTTCTCAGACC	GCCTGACCTGTTGCTTCAAT
SGK1	GTTCTGTCCCCATTGAGAGG	GGAGCCGATGGAGACTGATA
CoCl2 specific ERBS1	GGCTATCTTGACAGCGGGTC	TGGCAGGAAGCGAATTAGTCA
CoCl2 specific ERBS2	CCTCCGCCCTATGGGAAAAA	ATGTCGCTATGTGTTCTGGGA

Table S2. ChIP-sequencing statistics.

Sample	Type	Raw reads	Aligned reads	Unique positions	Normalization coefficient
**MCF7 Control EtOH**	Input	136,024,634	107,011,244	93,736,338	NA
**MCF7 Control E2**	Input	114,985,595	91,151,438	80,379,555	NA
**MCF7 Hypoxia EtOH**	Input	126,035,952	96,026,526	83,989,729	NA
**MCF7 Hypoxia E2**	Input	127,009,747	92,622,859	81,203,292	NA
**MCF7 Rescue EtOH**	Input	101,653,620	75,140,544	66,405,889	NA
**MCF7 Rescue E2**	Input	108,506,834	85,577,616	76,349,649	NA
**MCF7 Control EtOH**	ChIP ER	103,630,263	75,206,331	52,480,373	1.65
**MCF7 Control E2**	ChIP ER	156,709,777	123,215,788	83,214,383	1
**MCF7 Hypoxia EtOH**	ChIP ER	121,701,565	90,327,227	62,349,497	1.37
**MCF7 Hypoxia E2**	ChIP ER	90,330,137	62,648,747	39,791,861	1.98
**MCF7 Rescue EtOH**	ChIP ER	153,687,583	122,234,881	89,295,787	1.01
**MCF7 Rescue E2**	ChIP ER	119,442,446	90,309,476	65,287,259	1.36

Supplementary Table S3. Public dataset used.

		Transcription Factors	
**Cell line**	**Target**	**(Dataset)**	**Reference**
**MCF-7**	ESR1	/	Odom et al, 2010
	FoxA1_1	6566	Hurtado A, et al. Nat. Genet. 2011
	FoxA1_2	6567	Hurtado A, et al. Nat. Genet. 2011
	Dnase_1	45031	Maurano MT, et al. Science 2012
	Dnase_2	45032	Maurano MT, et al. Science 2012
	MAX	46324	Gertz J, et al.. Mol. Cell 2013
	GABPA	46314	Gertz J, et al.. Mol. Cell 2013
	REST	46313	Gertz J, et al.. Mol. Cell 2013
	SRF	46312	Gertz J, et al.. Mol. Cell 2013
	TAF1	46320	Gertz J, et al.. Mol. Cell 2013
	H3K4me3	UCSC dataset	Encode data
	H3K27ac	UCSC dataset	Encode data
	H3K27me3	UCSC dataset	Encode data
	H3K36me3	UCSC dataset	Encode data
	SIN3A	UCSC dataset	Encode data
	RAD21	UCSC dataset	Encode data
	PML	UCSC dataset	Encode data
	EP300	UCSC dataset	Encode data
	MAX	UCSC dataset	Encode data
	JUND	UCSC dataset	Encode data
	HDAC2	UCSC dataset	Encode data
	GATA3	UCSC dataset	Encode data
	Gabp	UCSC dataset	Encode data
	Foxm1	UCSC dataset	Encode data
	FosL2	UCSC dataset	Encode data
	Elf1	UCSC dataset	Encode data
	Egr1	UCSC dataset	Encode data
	Cebp	UCSC dataset	Encode data
	ZNF217	UCSC dataset	Encode data
	TCFL2	UCSC dataset	Encode data
	CTCF	UCSC dataset	Encode data

	TEAD4	UCSC dataset	Encode data
